# Recent Advances in Management by Pheromones of *Thaumetopoea* Moths in Urban Parks and Woodland Recreational Areas

**DOI:** 10.3390/insects10110395

**Published:** 2019-11-08

**Authors:** Pasquale Trematerra, Marco Colacci

**Affiliations:** Department of Agricultural, Environmental and Food Sciences, University of Molise, Via de Sanctis, I-86100 Campobasso, Italy

**Keywords:** *Thaumetopoea* complex, pheromones, monitoring, mass trapping, mating disruption

## Abstract

Caterpillars of the pine processionary moths, *Thaumetopoea* complex, cause serious defoliation to *Cedrus*, *Pinus,* and *Pseudotsuga* trees. *Thaumetopoea* caterpillars also have fine hairs on their abdominal tergites that contain a protein that can severely irritate and cause dermatitis in humans and domestic animals. The control of the *T. pityocampa* pine processionary moth has become necessary in many European countries because of the sanitary risks that are related to larval urtication and the defoliation threat to pine forests and plantations. New research activities have been aimed at the development of eco-friendly, innovative technologies for Integrated Pest Management (IPM) of these moths, particularly in urban parks and woodland recreational areas. This paper describes the recent advances in the use of pheromones in monitoring, mass trapping, and mating disruption related to management of processionary moths *T. hellenica* and *T. pityocampa*. According to the results, the use of pheromones may provide a practical alternative to insecticide sprays, as they can be safe and simple as compared to other control methods.

## 1. Introduction

The pine processionary moths, *Thaumetopoea* complex, are considered to be the most important insect defoliator of *Cedrus*, *Pinus,* and *Pseudotsuga* forests and woodlands in central-southern Europe and North Africa in terms of their geographic range and socioeconomic impact [1,2]. In particular, the temperature increases due to climate warming have favoured the expansion of *T. pityocampa* (Denis and Schiffermüller) into territories where it could not previously develop, including Switzerland, Germany, Austria, and England [3,4]. In the Mediterranean, *T. pityocampa* (present in Portugal, Spain, France, Italy, Balkan Peninsula except the Crete Island, Greece, Morocco, part of Algeria, and part of Turkey) occurs with *T. wilkinsoni* Tams (reported in Cyprus, Lebanon, part of Turkey, and Israel), and a clade provisionally named *T. pityocampa* ENA (cited in parts of Algeria, Tunisia, Libya), forming a species complex with distinct geographical distribution [4]. This clade has been recently identified in *T. hellenica* Trematerra and Scalercio, and *T. mediterranea* Trematerra and Scalercio, described from central Greece and Pantelleria Island, Italy, respectively [5].

In *T. pityocampa*, eggs are laid together in large numbers forming a sleeve around a pair of needles, the hatch time being 35–40 days. Larvae are present from July to March in a silken nest among branches; they leave nest at dusk to feed and then return at dawn. Larvae overwinter in a winter-nest emerging during February–April in a procession. Pupae are present in a cocoon below the ground. The adult flies at night during July–September. For more details regarding morphology, the biology and ecology of *Thaumetopoea* species, see Roques 2015 [6], and instead for *T. hellenica* and *T*. *mediterranea*, see Trematerra and Colacci 2018 [7].

**Figure 1:**
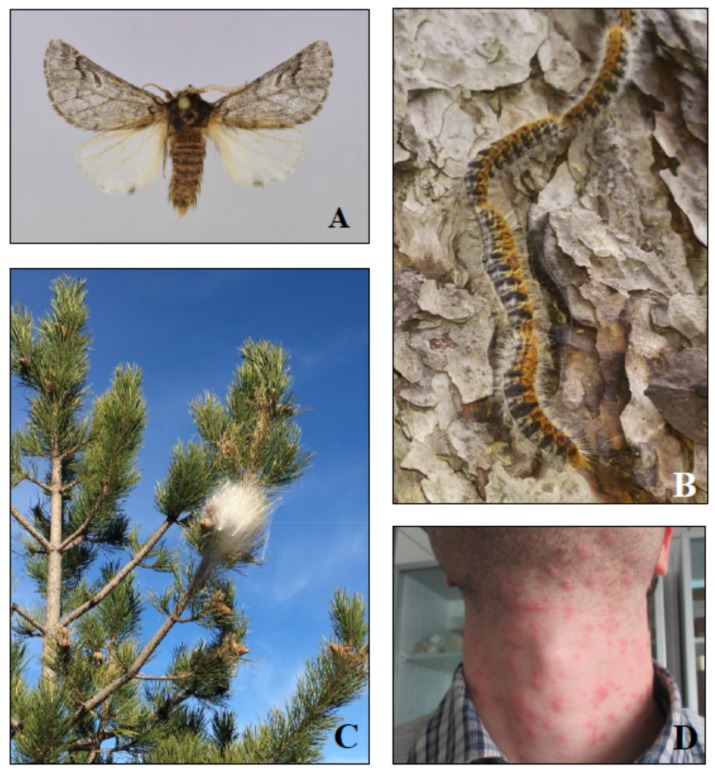
*Thaumetopoea pityocampa*: male (wingspan 30–40 mm) (**A**), mature larvae (about 40 mm) (**B**), winter nest (about 20 cm) (**C**) and urtication produced in humans (**D**).

The management of pine processionary moths, generally referred to as *T. pityocampa*, developed in Europe and in many other countries at the end of the 19th century, because of the risks associated with the defoliation threat to pine forests and plantations and the urticating larvae [8] (Figure 1). Currently, the most effective strategy for the control of these moths involves a combination of preventive techniques, such as planting policies and methods for early detection, and curative methods such as trapping of adults and larvae, elimination of winter nests, and the application of insecticides. Unfortunately, these methods may provide insufficient levels of control or endanger the health of human and domestic animals, particularly in urban parks and recreational suburban areas. Moreover, insecticide applications in the anthropized sites may be ineffective, as some parts of the plants remain untreated and spraying in inhabited areas often triggers complaints from residents. Biological control has to be stringently applied to achieve a satisfactory level of management [8,9,10,11].

In urban and suburban parks or in recreational areas, the use of pheromones can help to manage pine processionary moth infestations by aiding in the monitoring and direct control of male adults. In this paper, we report a review on the recent results that were obtained in the use of pheromones for monitoring, mass trapping, and mating disruption of *Thaumetopoea* complex [8,10,11,12,13,14,15,16,17].

## 2. Monitoring

Pheromone traps have been used in a wide variety of ways for insect pest management including seasonal phenology, population estimation, and decision support, as well as in early detection and the definition of invasive species.

The identification of “pityolure”, a potential sex pheromone of the pine processionary moth *T. pityocampa*, was performed in 1981 by Guerrero et al. [18]. Later, Camps et al. reported analogues of the sex pheromone with biological activity [19]. In 1997, Quero et al. [20] cited the component (*Z*)-13-hexadecen-11-ynyl acetate as the only female sex pheromone in the gland of *T. pityocampa*.

Cuevas et al. in Spain [21] and Einhorn et al. in the southwest of France [22] reported preliminary field trials on monitoring the pine processionary moth with the synthetic sex pheromone. Over several years, observations of the use of pheromone traps in the monitoring of *T. pityocampa* were conducted in north Italy by Tiberi and Niccoli [23]. Halperin also reported results of detection and control of the moth using pityolure [24] in Israel. More recently, preliminary results of the monitoring of the pine processionary moth in north and south Albania were reported by Horvath et al. [25], and by Athanassiou et al. [12] in Spain, Italy, and Greece.

Sex pheromone traps are now widely deployed and they have significantly contributed to the sustainable management of the moths. Despite the fact that there are some data available regarding the trapping of *T. pityocampa* males, there are few researches regarding the influence of a trap device on the capture of this species. The currently available traps and pheromones do not have the same efficiency in capturing male moths and they can be influenced by several factors, including the type of trap, trap position, pheromone, and visual stimuli, such as the colour of the trapping devices [2,26,27].

Jacktel et al. [2] compared the different pheromone trap devices for the capture of *T. pityocampa* males in France and in Portugal, and noted that traps with sticky surfaces were able to capture more adults than the non-adhesive ones on the basis of the mean number of captured males per trap device per day. Moreover, these data demonstrated that the increase of pheromone dose significantly augmented the number captured. Furthermore, sticky trap devices trapped significantly more males at the top of the tree crown as compared to lower heights. The authors [2] also cited that the mean numbers of males captured per day were significantly positively correlated with the number of winter nests per hectare. Another study observed that in Greece, the sticky trap devices Delta and Pherocon II types performed better than the Funnel traps in the capture of males [26]. There might be some certain drawbacks in the use of sticky Delta trap over the Funnel trap devices against *T. pityocampa*. Male adults have large bodies, which means that the sticky surface can be quickly saturated during catches. In comparison, Funnel traps are considered to be “high-capacity” trap devices [10]. These authors indicated that the pine density significantly affected the trap device performance and trap device colour should be considered to be one of the factors that can affect captures.

Given that different traps exhibit variable efficacy, the evaluation of novel trap devices in terms of their capture sensitivity and capacity could optimize the pheromone monitoring protocols of *T. pityocampa*. The use of large capacity traps that were baited with pityolure could facilitate the capturing of massive numbers of males to significantly decrease the mating probability. Athanassiou et al. [12] simultaneously evaluated six different trap devices in several different geographical locations: two sites for *T. hellenica* in Magnissia (Thessaly) and Attica, Greece; two sites for *T. pityocampa* in Campobasso, Italy, and Serra, Valencia, Spain. The first site in Greece was an area covered by 120 hectares of pines, mainly *Pinus brutia* Tenore and *P. halepensis* Miller. The second site was in Amarousion that included a *P. halepensis* wood. The site in Italy was in Petacciato, being covered by 35 ha of *P. halepensis* with *Pinus pinea* L. The site in Spain was in Porta Coeli, which is covered by 600 ha of *P. halepensis*.

**Figure 2:**
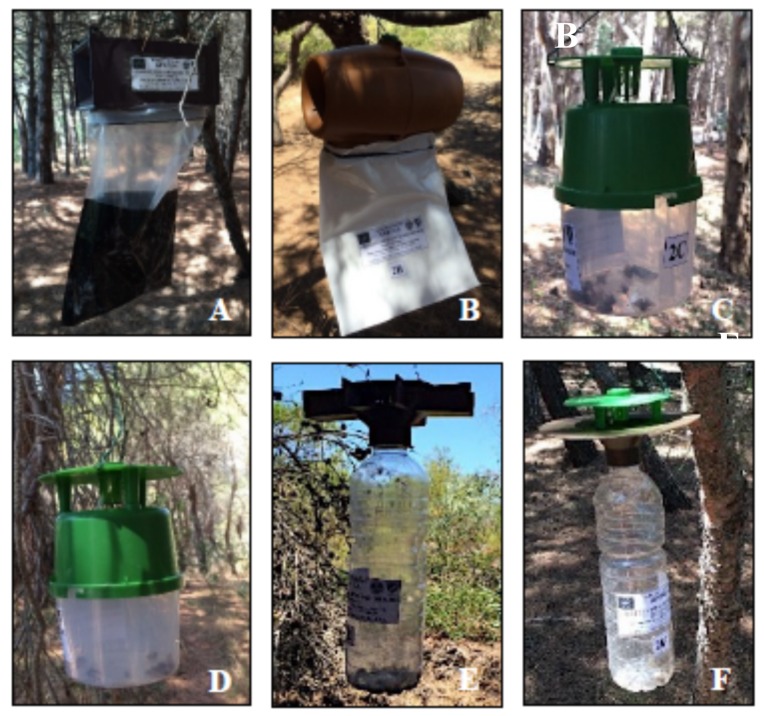
Funnel trap devices that were tested in trapping of *T. hellenica* and *T. pityocampa* male adults: G-Trap (**A**), Flysan (**B**), Lepisan (**C**), Lepisan plus (**D**), Prototype 5 (**E**), Prototype 6 (**F**).

Six trap devices, reported as Prototypes 1–6 in Athanassiou et al. [12], were employed for the trials: G-Trap (previously as Prototype 1) (SEDQ, Barcelona, Spain), Flysan (Prototype 2) (SanSan, Valencia, Spain), Lepisan (Prototype 3) (SanSan, Valencia, Spain), Lepisan plus (Prototype 4) (SanSan, Valencia, Spain), Prototype 5, and Prototype 6 (Figure 2). Athanassiou et al. report details of the traps [12].

All of the traps were baited with dispensers containing 1 mg of (*Z*)-13-hexadecen-11-ynyl acetate (Trécé Inc., Adair, Oklahoma). Lepisan plus contained two dispensers, each with 1 mg of the sex pheromone. In all sites, there were four parcels, with the exception of Attica, which had three parcels. The trap devices were suspended in early July to detect the initiation of male adult flight. During each weekly inspection, the traps were rotated clockwise within each parcel. Each trap was placed at a height of two to three metres from the ground. The pheromone dispenser was changed every four weeks. Athanassiou et al. reports statistical analyses and results [12].

In Goritsa Hill (Greece), the flight of *T. hellenica* started in early August and lasted until the end of October. The highest number of males was recorded during mid-September. Significantly more adults were captured in G-Trap (392 adults, 49.25% of the total trapped) than in the other traps. In Amarousion, Greece, the flight period started in mid-August and lasted until early November. Significantly more males were captured in G-Trap (299 adults, 49.50% of the total) than in the other traps. In Petacciato, Italy, the flight of *T. pityocampa* was initiated in early August and lasted until the first week of September. The highest number of males was recorded during early August. Significantly more adults were captured in G-Trap (801 adults, 48.84% of the total) than in the other trap devices. In Porta Coeli, Spain, the flight of *T. pityocampa* was initiated in early July and lasted until mid-September, but the vast majority of adults were trapped between late August and early September. Significantly more males were found in the G-Trap at this site (144 adults, 44.72% of the total).

**Figure 3:**
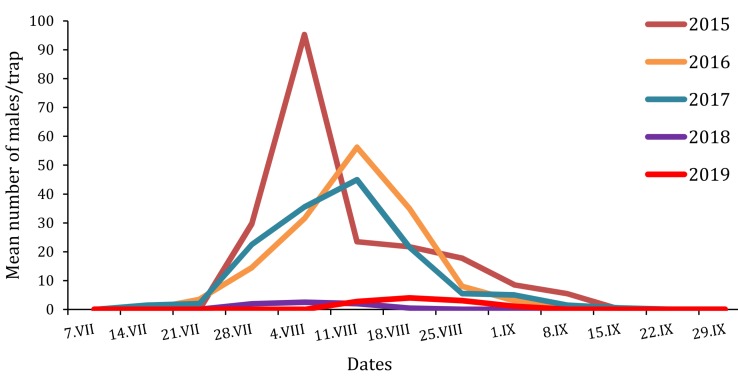
Flight phenologies of *T. pityocampa* males trapped in the Petacciato (Italy) recreational woodland area from 2015 to 2019.

Overall, the G-Trap was revealed as being superior to the other five traps used, with 2–12 fold increases in captured males when compared to other traps. While capture capacity is one of the most important elements of a given trap, the captures should be representative of the actual changes of male population densities in the test territory (Figure 3). Any asynchronies may lead to inaccurate conclusions and ineffective measures because the estimation of the peak period is one of the most important questions that need to be answered by the use of a trapping protocol. In this regard, trapping should be combined with additional sampling of the other life stages of *T. hellenica* and *T. pityocampa*, such as counting winter nests, in order to have a holistic and accurate view of its phenology.

**Figure 4:**
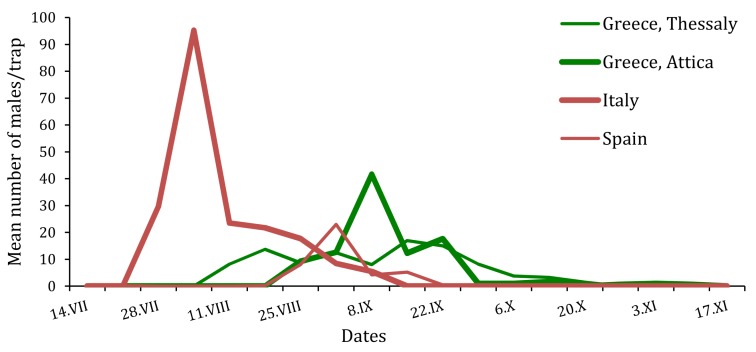
Flight phenologies of *T. hellenica* and *T. pityocampa* males trapped in the four experimental sites of Greece, Italy and Spain during the monitoring period 2015.

*T. hellenica* and *T. pityocampa* have different patterns of male adult catches in different areas (Figure 4). It is important to note these differences, as the accurate assessment of the seasonal abundance of the male adults is informative in order to time control measures, such as mass trapping and mating disruption for reducing the mating process. Furthermore, an accurate monitoring system of the male adult population will also provide valuable information for the application of rapid, degradable insecticides against larvae.

In Italy, during the monitoring of *T. pityocampa* on Monte Etna, and of *T. mediterranea* on Pantelleria Island, a different flight period of the adults was highlighted. *T. pityocampa* males started on 15 June and then lasted until the fall in September. *T. mediterranea* were trapped in the second week of July (14 July) and lasted until after the second week of October [7].

## 3. Experiments of Mass Trapping

The mass trapping techniques consist of using a relatively large number of traps to reduce population levels of the target species [28]. The traps can be baited with different types of semiochemicals or foods, acting on one or both sexes [29]. Mass trapping has been used to control many pests with different results; in some cases, it revealed a significant reduction in target pest population density or in pest damage. Several factors, such as trap competitiveness with wild females, trap design and density, population density, ecology of target pest, isolation, and risk of immigration can influence the success of this method [29,30,31].

For moth pest control, traps are baited with a sex pheromone that only attracts males; at least the trap devices must catch 80–95% of males in order to effectively reduce the infesting population [32]. Mass trapping against moths has been particularly employed in the following environments: in orchards against *Zeuzera pyrina* (L) and *Cossus cossus* (L.); in cotton and oilseed pests, such as *Spodoptera littoralis* (Boisduval) and *Pectinophora gossipiella* (Saunders); in stored products and food industries for *Ephestia* spp. and *Plodia interpunctella* (Hb.) [31]; and, in forests, for *Lymantria dispar* (L.) [30,33,34,35].

**Figure 5:**
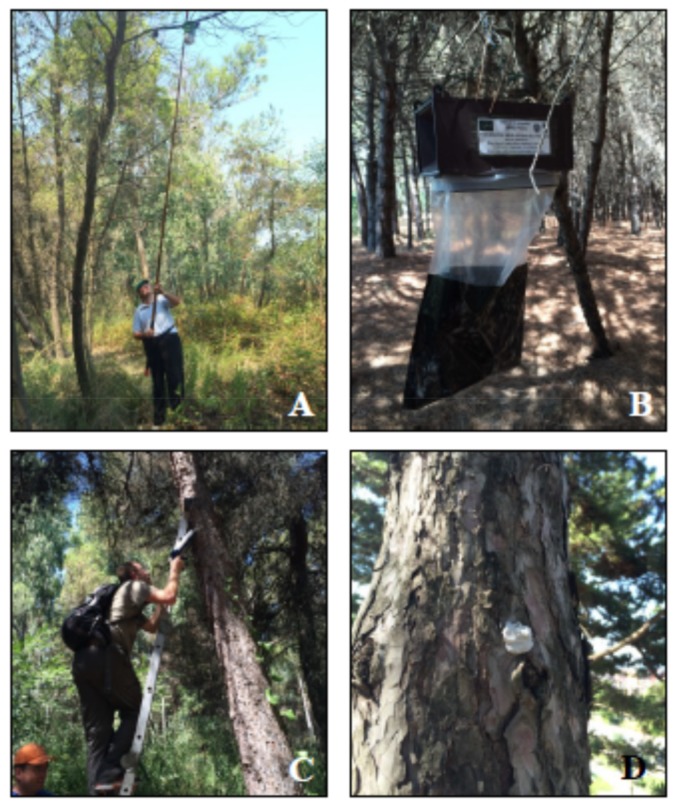
Control of processionary pine moths: positioning of pheromone trap in canopy of trees for monitoring and mass trapping tests (**A**–**B**) and application of pheromone paste on trunk and branch of trees for mating disruption trials (**C**–**D**).

Very little information is available regarding the mass trapping of *Thaumetopoea* moths. In 1983, initial field trials on mass trapping of *T. pityocampa* were carried out in a different part of Spain by Cuevas et al. [36] while using (*Z*)-13-hexadecen-11-ynyl acetate. Ten years later in Italy, Baronio et al. [37] reported an insignificant decrease in the number of *T. pityocampa* winter nests when compared to a control area after the distribution of 20 pheromone funnel-traps per hectare baited with pityolure. More recently, in 2015 Martin [8] from France reported that a minimum of four traps were necessary to be effective, even in a small site and six traps per hectare were needed for large sites. These numbers of traps are also consistent with the results that were obtained in the mass trapping of the moth in central Italy by Trematerra et al. [13]. These last authors experimented in a recreational coastal area of about 30 ha. The woodland was mainly composed of 50-year-old Aleppo trees, extending for 4 km along the Adriatic coast, with a width that ranged between 100 m and 350 m. During 2016, 2017, and 2018, two parcels of one hectare each were identified in the study area. The mass trapping method was applied for two years in one parcel (mass trapping parcel), whereas the other parcel was left untreated and it served as the control (control parcel). During 2016 and 2017, ten pheromone G-Trap types (SEDQ, Barcelona, Spain) were placed in the mass trapping-parcel to catch male adults (Figure 5).

The traps were baited with a plastic dispenser containing 1 mg of pityolure (Kenogard, Barcelona, Spain) and positioned in the canopy of plants approximately five to six metres above the ground. Four external monitoring population traps, which were spaced at one-kilometre intervals, were placed throughout 2015. The winter nests realized by overwintering larvae of *T. pityocampa* in the mass trapping parcel and control parcel were noted while using binoculars. Statistical analyses and results are reported in Trematerra et al. [13].

After two years of mass trapping, 53% and 79% fewer adults were collected in the mass trapping parcel compared to the control parcel, respectively, in the first and second year of the experiment (2016 and 2017). The total number of male moth adults collected in the control parcel was 353 in 2015, 426 in 2016, 476 in 2017, and 14 in 2018. In the mass trapping parcel were collected 131 in 2016, 56 in 2017, and 13 in 2018. In 2016, the average number of winter nests per Aleppo tree in the mass trapping parcel and control parcel were similar prior to applying the mass trapping method, approximately 0.71 and 0.87 nests/tree, respectively. In 2017, after one year of mass trapping, the average number of winter nests per tree in the mass trapping parcel (0.08 nests/tree) was decreased when compared to the control parcel (0.50 nests/tree). Similar results were obtained in 2018, after two years of mass trapping, with 0.04 nests/tree being present in the mass trapping parcel vs. 0.16 nests/tree being present in the control parcel. Trematerra et al. [13] reported that, at a density of 10 traps/hectare, mass trapping in mass trapping parcels reduced nests by 88% after one year and 94% after two years, when compared to the nest reduction of 43% and 80% observed in the control parcel. These preliminary results may have also been facilitated by the low mobility of *Thaumetopoea* females and the landscape configuration of the experimental forest, which reduced the immigration rate to the lateral sides of the parcels [38].

Trap density might reflect a compromise between the density that delivers the highest catch per unit area and the one that is the most cost-effective. The selected trap density should be periodically reviewed as pest population, scale of operation, and improvements in trap design and lure performance all work to reduce the number of traps that are needed to achieve economically acceptable levels of control.

## 4. Experiments of Mating Disruption

For mating disruption, it has been established that, in Lepidoptera, synthetic female pheromone is released in a treated site to compete with natural pheromone that is produced by calling females, and it can drastically reduce mate-finding by males and thus mating in general [17,39]. To date, mating disruption is the most developed pheromone-based technology for the direct control of moth pests. In general, it has been successfully evaluated and commercialized for a very wide diversity of moths of economic importance, including *Lobesia botrana* (Den. & Shiff.), *Cydia pomonella* (L.), *Grapholita molesta* (Busck), *Ephestia* spp., *Plodia interpunctella* (Hb.), and *Cossuss insularis* (Staudinger) [40,41,42,43]. There are disproportionately few data for moth species that are important pests of forest trees due to the lower cost efficiency of a mating disruption based strategy applied on a large scale, remembering that in the Gypsy moth, this tactic is only effective against low level populations [44,45].

Few studies exist regarding the use of this system for the management of pine processionary moths. Univoltine species, like *Thaumetopoea,* might be ideal for mating disruption, as a single annual application might rapidly lead to population suppression without the need for reapplication. However, the timing of the application is critical. Trials on mating disruption against *T. pityocampa* have been mostly limited to pilot studies that were carried out several decades ago in small areas of Israel and Italy [24,46]. During 2004 and 2005, data were obtained in France by Martin and Frérot [47]. New observations on mating disruption were reported from Italy and Greece by Trematerra et al. [14]. In this framework, the objective was to evaluate mating disruption for the control of *T. hellenica* and *T. pityocampa* for two consecutive years. Trials were conducted targeting *T. hellenica* from Summer 2015 to Winter 2017 in two sites in Greece, Goritsa Hill, and the Institute of Agricultural Sciences (IAS), and targeting *T. pityocampa* in one site in Petacciato, Italy.

In both years, traps that were baited with dispensers containing 1 mg of pityolure were suspended on pine branches to monitor adult male *Thaumetopoea* flight activity before and after mating disruption application and to determine the beginning of the flight period. The mating disruption parcels were treated with pityolure that was formulated in paste (ThauPi-polymix, 2% active ingredient, Novagrica Hellas, Athens, Greece). A 250 g syringe operated with a standard caulk gun was used to apply pheromone paste in small drops of approximately 2.5 g (Figure 5). The total concentration of pityolure applied in each parcel was 20 ± 0.5 g active ingredient/ha. Pheromone traps were checked at regular intervals for monitoring of adult male flight activity before and after mating disruption application. Additionally, in winter 2016 and 2017, the winter nests that had been created by *T. hellenica* and *T. pityocampa* larvae on colonized pine trees of the experimental parcels were counted via observation. Trematerra et al. reported statistical analyses and results [14].

During the two years of experiment, the reduction in trap catches and winter nests was similar in Greece and Italy. For Goritsa Hill and in IAS, the total number of male moths of *T. hellenica* that were trapped in mating disruption parcels after pheromone application was significantly lower than that in the control parcels during the same interval. In Goritsa, the total number of males collected in the control parcel was 19 in 2015 and 474 in 2016; in the mating disruption parcel, it was three in 2015 and five in 2016. In IAS, the number of male moths collected in the control parcel was 1446 in 2015 and 1515 in 2016; in the mating disruption parcel, it was 272 in 2015 and 53 in 2016. In Petacciato, for *T. pityocampa*, the number of males that were captured in the mating disruption parcel after pheromone application was significantly lower than that in untreated parcels during the same interval. The total number of males that were collected in the control parcel was 353 in 2015 and 400 in 2016; in the mating disruption parcel, it was 49 in 2015 and 35 in 2016 [14].

The mean number of winter nests for each site was also significantly lower in the mating disruption parcels when compared with the control parcels. In Goritsa, the total number of winter nests counted in the control parcel was 235 in 2016 and 564 in 2017; in the mating disruption parcel, it was 50 in 2016 and 14 in 2017. In IAS, the total number of winter nests counted in the control parcel was 71 in 2016 and 59 in 2017; in the mating disruption parcel, it was 18 in 2016 and eight in 2017. In Petacciato, the total number of winter nests counted in the control parcel was 21 in 2016 and 12 in 2017; in the mating disruption parcel, it was three in 2016 and one in 2017.

According to the results that were reported by Trematerra et al. [14], mating disruption is able to disrupt the mating behaviour and reproductive success of a large proportion of the population of *T. hellenica* and *T. pityocampa*, which clearly suggests that this method can be used for the control of these species. It should be noted that trap shut-down is not a reliable indicator for the success of this method [39]; evaluating the number of winter nests in the treated and untreated sites confirmed the efficacy of mating disruption.

The incorporation of mating disruption into programmes for control of processionary pine moths will require a greater understanding of how mating disruption works and a better refinement of how it can be synergistically combined with other pest management tactics [17].

## 5. Conclusions

Strategies for the management of the *Thaumetopoea* complex, pine processionary moths, include a combination of preventive techniques, such as planting policies and methods for early detection, and curative methods, such as the removal of egg batch nests, trapping of migrant larvae, spraying microbials, including *Bacillus thuringiensis* var. *kurstaki* Berliner or IGR insecticides, bio-control, mass trapping, and mating disruption of adult males.

The mechanical removal of egg batches or the nests and the use of trunk barrier traps, usually applied tree by tree, are feasible for small stands, but difficult to apply over large areas. Mechanical removal techniques have the disadvantage of not being exhaustive, as visualizing the egg batches and the autumn nests in the canopy is difficult. The winter nests are clearly visible, but it is often difficult to reach them when they are in the upper parts of tree canopies.

Trunk barrier traps can be used in late winter or early spring to capture migrant larvae as they descend from the winter nests. Martin et al. [10] reported the high efficiency (96.5%) of the trap device Ecopiege; similarly, Colacci et al. [48] noted the high capture capacity of the trunk barrier trap devices Procerex and LIFE-PISA prototype (with efficiency of 99.5% and 98%, respectively). Deploying one trunk barrier trap per plant is not practical over large areas as in a forest, despite these results.

The method of spraying the tree canopy with *B. thuringiensis* has been in use since 1980 [49]. The effectiveness of this method is particularly high against the caterpillar of *T. pityocampa* and it has been tested in several experiments [50,51,52,53]. Another group of biocides used for the control of the processionary larvae with interesting results are the insect growth regulators (IGRs) [54]. However, under forest conditions, chemical treatments that were carried out by air or surface vehicles are expensive and sometimes impractical due to the orography.

When compared to other pest control methods, the use of pheromones in mass trapping and mating disruption might provide a practical alternative to insecticide sprays, particularly in urban and suburban parks, public or private gardens, and recreational areas. Mass trapping is cost effective when compared to mating disruption, as much smaller quantities of pheromones are required [55]. Future control methods may include the trapping and control of females by natural semiochemicals [56,57,58]. New data are required for attract-and-kill or attract-and-infect technologies (see Allison and Cardé [16]).

It should be noted that pheromone use does not limit the possibility of re-infestation by female processionary pine moths that come from the surrounding areas and, for this reason, effectiveness is greater in the isolated sites and during long-term application [59,60,61,62].

In the context of IPM, according to the reported results, mass trapping and mating disruption can be adopted in combination with preventive techniques and other control methods. Moreover, the application of pheromones against *Thaumetopoea* moths could be justifiable for urban and suburban parks, private gardens, and recreational areas in terms of economic viability and ease of application. This is particularly important while considering the risks to public health and domestic animals. In contrast, given that pine is a forest tree that is abundant in the Mediterranean and forests of northern Europe, an area-wide application of mass trapping and mating disruption for the control of these species might not be viable, as human resources access in all target areas may be limited and the cost of the application may be high.
